# Reactive Disruption of the Hippocampal Neurogenic Niche After Induction of Seizures by Injection of Kainic Acid in the Amygdala

**DOI:** 10.3389/fcell.2019.00158

**Published:** 2019-08-20

**Authors:** Teresa Muro-García, Soraya Martín-Suárez, Nelson Espinosa, Roberto Valcárcel-Martín, Ainhoa Marinas, Laura Zaldumbide, Lara Galbarriatu, Amanda Sierra, Pablo Fuentealba, Juan Manuel Encinas

**Affiliations:** ^1^The Neural Stem Cell and Neurogenesis Laboratory, Achucarro Basque Center for Neuroscience, Leioa, Spain; ^2^Department of Neurosciences, University of the Basque Country (UPV/EHU), Leioa, Spain; ^3^Departamento de Psiquiatría, Centro Interdisciplinario de Neurociencia, Pontificia Universidad Católica de Chile, Santiago, Chile; ^4^Epilepsy Unit, University Hospital of Cruces, Bilbao, Spain; ^5^Laboratory of Glial Cell Biology, Achucarro Basque Center for Neuroscience, Leioa, Spain; ^6^IKERBASQUE, The Basque Foundation for Science, Bilbao, Spain

**Keywords:** neural stem cells, hippocampal neurogenesis, seizures, gliosis, amygdala

## Abstract

Adult neurogenesis persists in the adult hippocampus due to the presence of multipotent neural stem cells (NSCs). Hippocampal neurogenesis is involved in a range of cognitive functions and is tightly regulated by neuronal activity. NSCs respond promptly to physiological and pathological stimuli altering their neurogenic and gliogenic potential. In a mouse model of mesial temporal lobe epilepsy (MTLE), seizures triggered by the intrahippocampal injection of the glutamate receptor agonist kainic acid (KA) induce NSCs to convert into reactive NSCs (React-NSCs) which stop producing new neurons and ultimately generate reactive astrocytes thus contributing to the development of hippocampal sclerosis and abolishing neurogenesis. We herein show how seizures triggered by the injection of KA in the amygdala, an alternative model of MTLE which allows parallel experimental manipulation in the dentate gyrus, also trigger the induction of React-NSCs and provoke the disruption of the neurogenic niche resulting in impaired neurogenesis. These results highlight the sensitivity of NSCs to the surrounding neuronal circuit activity and demonstrate that the induction of React-NSCs and the disruption of the neurogenic niche are not due to the direct effect of KA in the hippocampus. These results also suggest that neurogenesis might be lost in the hippocampus of patients with MTLE. Indeed we provide results from human MTLE samples absence of cell proliferation, of neural stem cell-like cells and of neurogenesis.

## Introduction

In the hippocampus of most mammals, including humans (Eriksson et al., 1998; Moreno-Jiménez et al., 2019), neurogenesis continues postnatally (Altman and Das, 1965) and throughout adulthood due to the existence of a population of neural stem cells (NSCs) with neurogenic (Seri et al., 2001) and gliogenic potential (Encinas et al., 2011). Neuronal activity is a major regulator of adult hippocampal NSCs which respond differentially to different levels of neuronal activity. Tonic gamma-aminobutyric acid (GABA) promotes quiescence of NSCs while its reduction promotes their activation (entry into the cell cycle) (Song et al., 2012). As neuronal activity increases above physiological levels in experimental models of electroconvulsive therapy (electroconvulsive shock, ECS) (Segi-Nishida et al., 2008; Jun et al., 2015) or epilepsy (Huttmann et al., 2003; Indulekha et al., 2010) NSCs get activated in increasing numbers which in turn accelerates their depletion in the long term (Sierra et al., 2015) after an initial boost of neurogenesis (Parent et al., 1997). Furthermore, in an experimental model of mesial temporal lobe epilepsy (MTLE), that characterizes by seizures being originated in the hippocampus and related structures, NSCs undergo a profound alteration of their neurogenic program. Shortly after seizures NSCs transform into reactive NSCs (React-NSCs) and later into reactive astrocytes that contribute to hippocampal sclerosis, a pathological hallmark of MTLE consisting of neuronal death and reactive gliosis. React-NSCs get activated massively switching to a symmetric manner of cell division and transform into a reactive-like multibranched and thickened phenotype with overexpression of nestin and GFAP (Sierra et al., 2015). As a result neurogenesis is lost. The rodent MTLE model is based on a single injection of the glutamate receptors agonist kainic acid (KA) in the hippocampus (hcMTLE) (Bouilleret et al., 1999). Thus it could be argued that the strong effects observed in the neurogenic niche could be due to the direct effect of KA. Thus we wondered how NSCs and the neurogenic cascade would be affected in an alternative model of MTLE (aMTLE) based on the intra-amygdalar injection of KA (Mouri et al., 2008). In addition, we showed that in a similar fashion to what we found in the mouse models of MTLE, cell proliferation and neurogenesis are lost in the hippocampus of MTLE patients.

## Materials and Methods

### Animals

Nestin-GFP transgenic mouse line (on a C57BL/6 background) was used for all procedures addressing tissue analysis (Sierra et al., 2015) except for three wild type mice used for the injection tracking (see below). At least three mice were used in each control (PBS) group and at least five were used for the aMTLE group per time point. All procedures on these mice were approved by the University of the Basque Country (EHU/UPV) Ethics Committee (Leioa, Spain) and the Comunidad Foral de Bizkaia (CEEA: M20/2015/236). C57BL/6 mice were used for the EEG studies. The procedures were approved by the Comité Ético Científico para el Cuidado de Animales y Ambiente, CEC-CAA of the Universidad Pontificia de Chile and the bioethics committee of the Chilean (Comision Nacional de Investigación Científica y Tecnólogica, CONICYT). Five mice were used in each control (PBS) and aMTLE group.

### Intra-Amygdalar Injection

**FIGURE 1:**
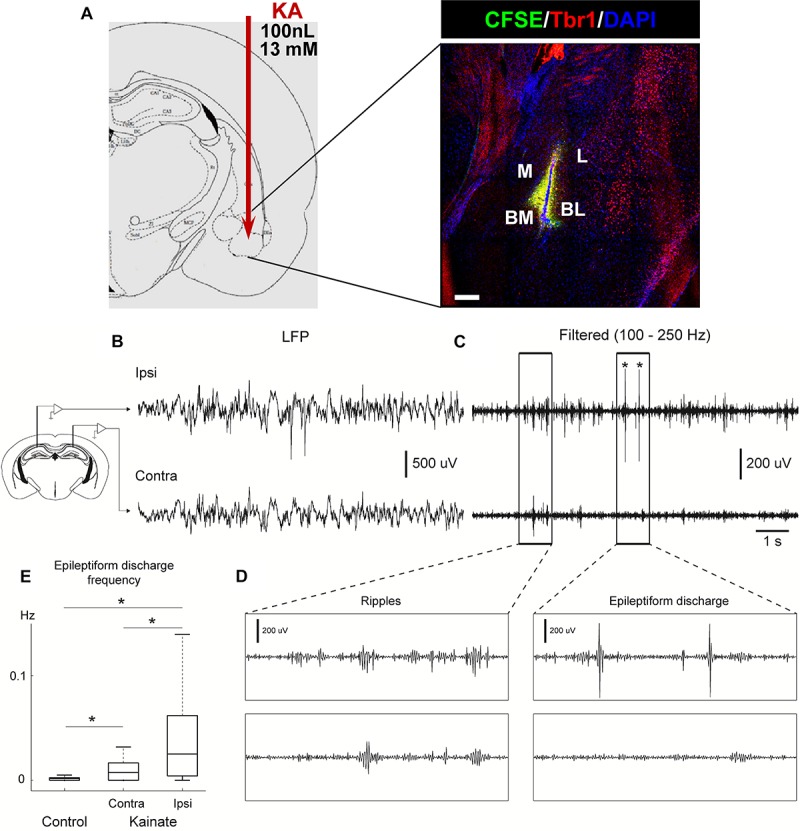
Intra-amygdalar injection of KA induces seizures and epileptiform activity in the ipsi and contralateral hippocampus. **(A)** KA was stereotaxically injected via canula into the basomedial basolateral nucleus of the amygdala (BM/BL, 100 nL at a concentration of 13 mM). The accuracy of the injection was tested by injecting fluorescein (CFSE) and sacrificing mice 24 h later. Co-staining with Tbr1 and DAPI was performed to assure identification of the BL. M, medial nucleus. BM, basomedial nucleus. L, lateral nucleus. CEP, endopiriform cortex. Scale bar is 50 μm in **(A)**. **(B)** Intrahippocampal electrophysiological recordings performed 2 months after KA injection. Local field potential (LFP) activity recorded in the hippocampal pyramidal layer from one electrode in the ipsilateral hemisphere (KA injected side, Ipsi, upper panel) and one electrode in the contralateral hippocampus (uninjected side, Contra, lower panel) in a urethane anesthetized mouse. **(C)** Filtered LFP (100–250 Hz). Asterisks depict epileptiform discharges. **(D)** Zoomed in activity depicts wave ripple complex during basal activity (left) and epileptiform discharge (right) for filtered signal shown in **(C)**. **(E)** Epileptiform discharge events were automatically detected in both control and KA mice (see section “Supplementary Material” for details). Epileptiform events were more abundant in KA mice (*n* = 52 sessions, 5 animals) compared to control mice (*n* = 33 sessions, 5 animals). Moreover, in KA mice, the hemisphere ipsilateral to KA injection (Ipsi) had a higher rate of epileptiform events compared to contralateral (Contra) hemisphere (Kruskal–Wallis test, *P* = 1.049 × 10^–7^).

PBS or KA was injected into the right basolateral-amygdala using the following coordinates: AP −1.4 mm, ML −3.1 mm, and DV −4.7 mm, using a dose of 100 nL of sterile PBS, or KA at 13 mM (1.3 nmol). Three mice were injected with fluorescein (CSFE) and sacrificed 24 h after to test the accuracy of the stereotaxic injection (Figure 1).

### Electroencephalographic Recordings

Neuronal activity was recorded by using a 32-channel silicon probe (Right hemisphere: A1 × 32-Poly3-6 mm-50-177; left hemisphere; 32 channel-4 shank silicon probe, Buzsáki 32. Neuronexus, mean resistance 1 MΩ) stained with DiI (for a subsequent anatomical identification). Electrodes were located as close as possible to the dorsal CA1 stratum pyramidale, for which the electrode was descended 0.8–1 mm, until ripple oscillations were visually detected online. Electrical activity was recorded with an electrical amplifier (Intan RHD 2132 amplifier board connected to an RHD2000 evaluation system; Intan Technologies). Local field potential (LFP; sampling rate 20 kHz) were digitally filtered between 0.3–2 kHz.

### 5-Bromo-2′-deoxyuridine (BrdU) Administration

BrdU was administered intraperitoneally (four injections 2 h-apart)on the 2nd day after the intra-amygdalar injection (four injections 2 h-apart).

### Immunohistochemistry and Cell Quantification

Experiments were performed essentially as described before following methods optimized for the use in transgenic mice (Encinas et al., 2006, 2011; Encinas and Enikolopov, 2008; Sierra et al., 2015).

### Image Capture

All fluorescence immunostaining images were collected employing a Leica SP8 laser scanning microscope and their corresponding manufacturer’s software following protocols optimized for stereotaxic quantification and quantitative image analysis (Encinas et al., 2011; Sierra et al., 2015).

### Human Tissue

Human samples from individuals with MTLE. Freshly resected hippocampi from adult drug-resistant MTLE patients were obtained from the Basque Biobank at the Cruces University Hospital (Bilbao, Spain) with the patient’s written consent and with approval of the University of the Basque Country Ethics committee (CEISH/154/2012).

### Statistical Analysis

SigmaPlot (San Jose, CA, United States) was used for statistical analysis. For the analysis of pairs of groups a Student’s *t*-test, a Mann–Whitney Rank Sum test or a One-column sample test were performed.

For an extended description of see sections “Materials and Methods” and “Supplementary Material and Methods” in Supplementary Material.

## Results

We first confirmed the accuracy of the coordinates used to target the basolateral nucleus of the amygdala by injecting fluorescein (CFSE) and co-staining for DAPI and the neuronal marker Tbr1 to assure identification of the exact area (Figure 1A). Then we analyzed the effect on neuronal activity of the intra-amygdalar injection of KA to confirm its validity as a model of MTLE to study alterations of hippocampal neurogenesis. In hcMTLE a single injection is enough to trigger an initial set of seizures and then spontaneous seizures become chronic (Bouilleret et al., 1999; Sierra et al., 2015). Here we confirmed the existence of initial seizures behaviorally (using the Racine scale) on the day of the surgery and then registered neuronal activity by electroencephalographic recordings (EEG) through bilateral electrode insertion in the hippocampus (Figure 1B and Supplementary Figure 1) at a later time point. Seizures (Figure 1B), as well as ripples (Figure 1C) and epileptiform discharges (Figure 1D), were registered in both the ipsi and contralateral hippocampus of aMTLE mice 2 months after the KA injection. The frequency of the epileptiform activity was quantified by automatic detection of epileptiform events set up using baseline activity under anesthesia in control (PBS-injected) and aMTLE mice (Supplementary Figure 1). The frequency of the epileptiform events was significantly higher in the contralateral hippocampus of aMTLE mice (*n* = 5) than in controls (ipsilateral, *n* = 5). In the ipsilateral hippocampus of aMTLE mice (*n* = 5) epileptiform events were significantly more frequent than in the control and the contralateral aMTLE hippocampus (Figure 1E).

**FIGURE 2:**
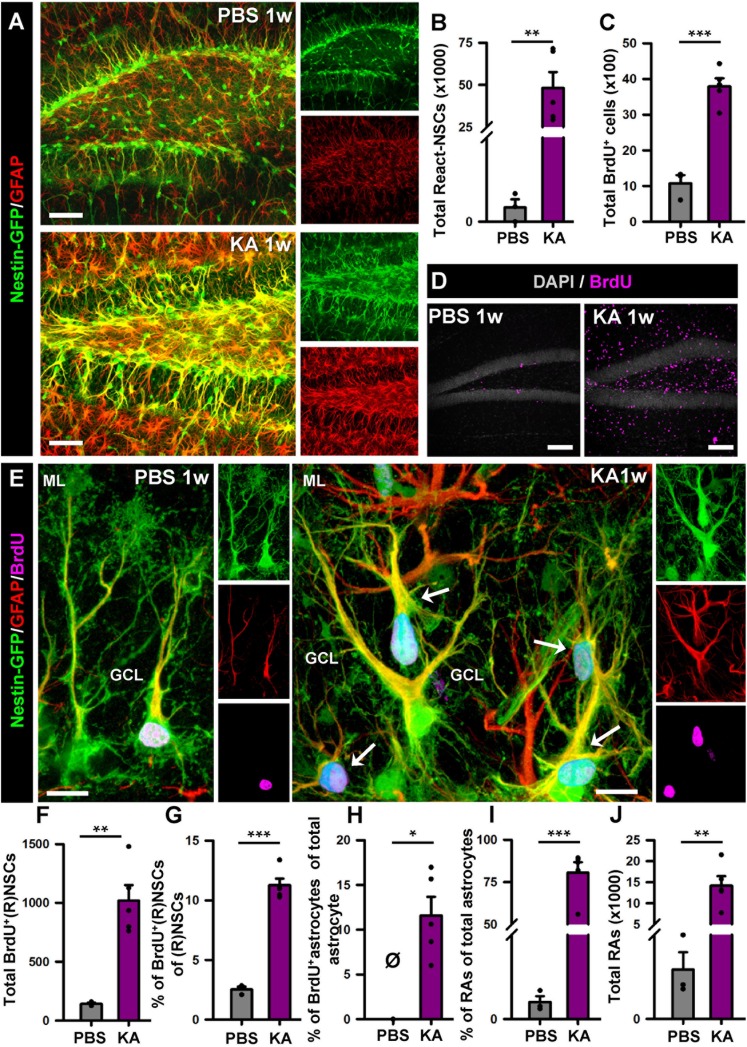
Intra-amygdalar injection of KA induces gliosis and React-NSCs in the dentate gyrus in the short term (1 week). BrdU (four injections, 2 h-apart) was injected the 2nd day after the KA injection. **(A)** Representative confocal microscopy images showing the reactive gliosis developed in the dentate gyrus. **(B)** Quantification of the number of React-NSCs (Nestin-GFP/GFAP-positive, S100ß-negative cells with reactive morphology). **(C)** Quantification of the number of BrdU-positive cells. **(D)** Representative confocal microscopy images for BrdU staining. **(E)** Representative confocal microscopy images showing a NSC in a control mouse and React-NSCs in a KA mouse labeled with BrdU (arrows). **(F–I)** Quantification of: **(F)** total number of BrdU-labeled NSCs or React-NSCs (Nestin-GFP/GFAP-positive, S100ß-negative); **(G)** percentage of BrdU-label NSCs or React-NSCs among the total number of NSCs/React-NSCs; **(H)** percentage of BrdU-labeled astrocytes (BrdU/GFAP-positive, Nestin-GFP-negative cells) among the total number of astrocytes (GFAP-positive Nestin-GFP-negative); **(I)** percentage of reactive astrocytes (Nestin-GFP/S100ß-positive cells) among the total number of astrocytes; and **(J)** total number of reactive astrocytes. All quantifications are referred to the SGZ + GCL. For quantifications regarding the hilus refer to (Supplementary Figure 3). Scale bar is 50 μm in **(A)** and **(D)**; and 10 μm in **(E)**. *n* = 3 for PBS and 5 for KA mice. ^∗∗∗^*p* < 0.001, ^∗∗^*p* < 0.005, ^∗^*p* < 0.05 by Student’s *t*-test **(B,C,G)**; One-column sample *t*-test **(H)** and Mann–Whitney Rank Sum test **(I,J)**. Bars show mean ± SEM. Dots show individual data. The same settings for image acquisition were used for PBS and KA samples.

**FIGURE 3:**
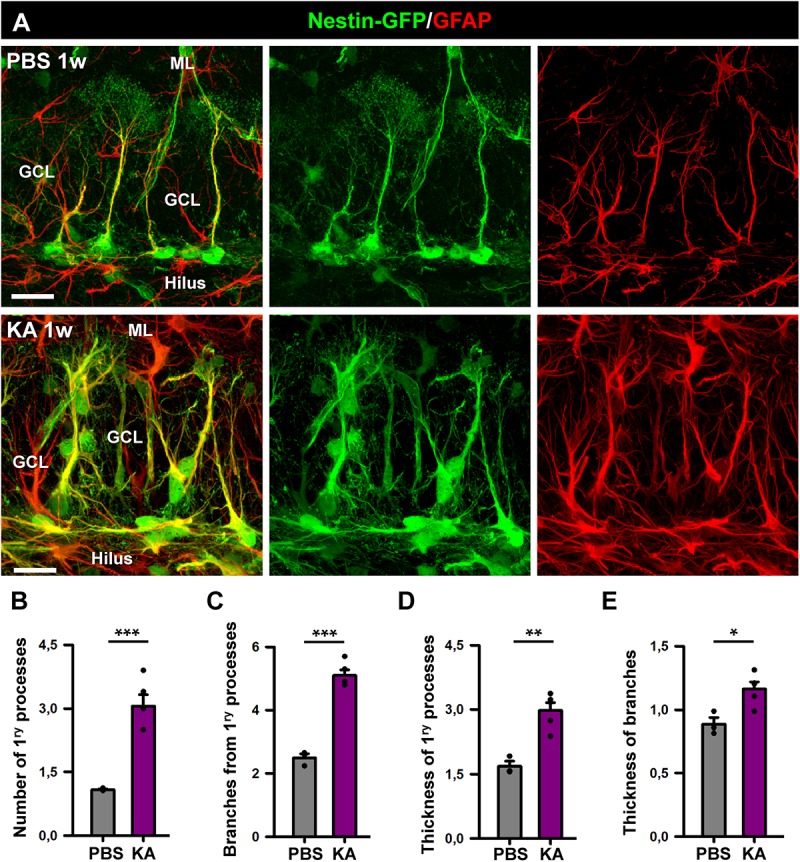
Morphological parameters of React-NSCs. **(A)** Confocal microscopy images of NSCs in a control mouse (upper row) and React-NSCs in a KA mouse (lower row). **(B–E)** Quantification of number of primary processes (those directly emerging from the soma) **(B)**; number of secondary processes (those emerging from the primary processes) **(C)**; thickness of the primary process **(D)**; and secondary processes **(E)**. *n* = 5 per group. ^∗∗∗^*p* < 0.001, ^∗∗^*p* < 0.005, ^∗^*p* < 0.05 by Student’s *t*-test. Bars show mean ± SEM. Dots show individual data.

**FIGURE 4:**
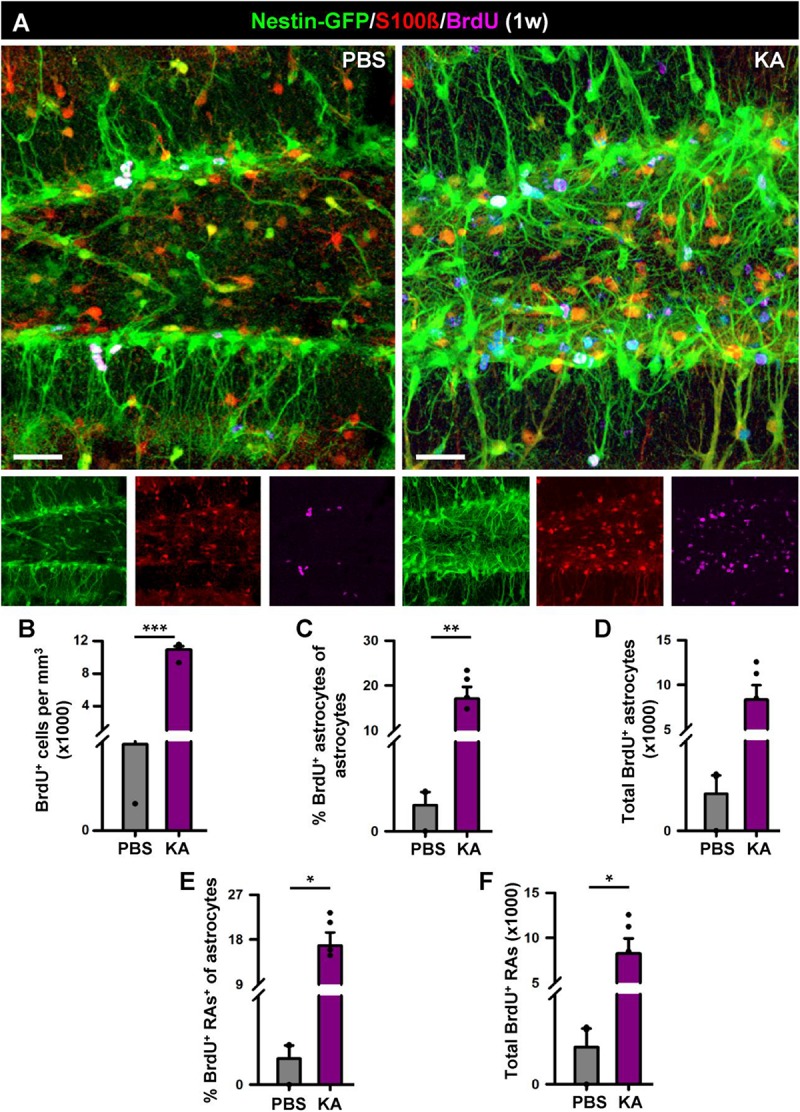
Development of reactive gliosis in the hilus in the short term (1 week). **(A)** Representative confocal images of PBS (left panel) and KA (right) mice after staining for Nestin-GFP, BrdU and S100β to assess reactive gliosis. **(B–F)** Quantification of the density of BrdU^+^ cells **(B)**; percentage of BrdU-labeled astrocytes among astrocytes **(C)**; total number of BrdU-labeled astrocytes **(D)**; percentage of BrdU-labeled of reactive astrocytes among astrocytes **(E)**; total BrdU-labeled reactive astrocytes **(F)**. Scale bar is 20 μm. *n* = 5 per group. ^∗∗∗^*p* < 0.001, ^∗∗^*p* < 0.005, ^∗^*p* < 0.05 by Student’s *t*-test **(C,E)** and Mann-Whitney Rank Sum test **(B,D,F)**. Bars show mean ± SEM. Dots show individual data.

**FIGURE 5:**
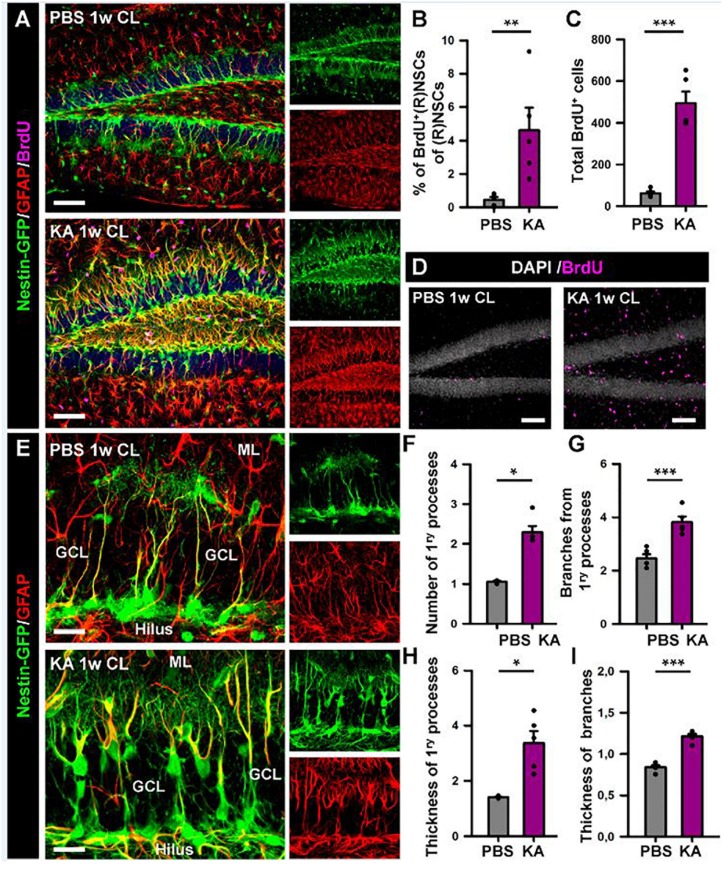
Intra-amygdalar injection of KA induces gliosis and React-NSCs in the contralateral dentate gyrus. BrdU (four injections, 2 h-apart) was injected the second day after the KA injection. Animals were sacrificed 1 week after the PBS or KA injection. **(A)** Representative confocal microscopy images showing the reactive gliosis developed in the dentate gyrus. **(B)** Quantification of the percentage of BrdU-label NSCs or React-NSCs among the total number of NSCs/React-NSCs. **(C)** Quantification of the total number of BrdU-positive cells in the SGZ + GCL. **(D)** Representative confocal microscopy images for BrdU staining. **(E)** Confocal microscopy images of NSCs in a control mouse (upper row) and React-NSCs in a KA mouse (lower row). **(F)** Quantification of number of primary processes (those directly emerging from the soma); **(G)** Number of secondary processes (those emerging from the primary processes); **(H)** thickness of the primary process; and **(I)** secondary processes. *n* = 5 per group. Scale bar is 50 μm in **(A)** and 10 μm in **(E)**. ^∗∗∗^*p* < 0.001, ^∗∗^*p* < 0.005, ^∗^*p* < 0.05 by Student’s *t*-test **(B,G,I)** and Mann–Whitney Rank Sum test **(C,F,H)**. Bars show mean ± SEM. Dots show individual data.

We proceeded to analyze the dentate gyrus of mice injected either with PBS or KA in the amygdala and sacrificed 1 week later (Figure 2). We observed an overall overexpression of GFAP and Nestin-GFP typical of reactive gliosis (Figure 2A). The number of React-NSCs, as previously described for hcMTLE (Sierra et al., 2015), was drastically increased in the aMTLE mice compared to the PBS-injected mice (Figure 2B). React-NSCs presented several thickened prolongations emerging from the soma, had lost the fine broccoli-like apical arborization, overexpressed Nestin-GFP and GFAP and frequently moved into the granule cell layer (GCL) away from the subgranular zone (SGZ) (Figure 2E). Quantification of these morphological parameters is shown in Figure 3. To evaluate cell proliferation we administered BrdU on the second day (four injections, 2 h apart) after the intra-amygdalar injection of KA or PBS. The overall number of BrdU-labeled cells was significantly increased in the SGZ + GCL (Figures 2C,D) as well as in the hilus (Figures 4A,B) where astrocytes (GFAP-positive, Nestin-GFP-negative cells) and reactive astrocytes (Nestin-GFP/S100ß-positive cells) accounted for most of the BrdU-labeled cells (Figures 4C–F). As expected, BrdU incorporation by React-NSCs (Nestin-GFP/GFAP-positive and negative for S100ß) was significantly higher than their normal NSCs counterparts of the PBS-injected mice (Figures 2E–G) both in percentage (Figure 2F) and in total numbers (Figure 2G). Astrocyte proliferation was almost absent in the PBS animals but was increased in the KA mice (Figure 2H). We quantified, also in the SGZ + GCL, the presence of reactive astrocytes (Nestin-GFP/S100ß-positive cells, Supplementary Figure 2) to evaluate the development of gliosis in the dentate gyrus. Reactive astrocytes were mostly absent in the PBS mice whereas their proportion among total astrocytes (Figure 2I) as well as their total number (Figure 2J) increased in the aMTLE mice. Together these results show the early transformation of NSCs into React-NSCs in response to the local neuronal hyperexcitation triggered by the intra-amygdalar injection of KA (Figure 5E). Furthermore, we confirmed the induction of React-NSCs (Figure 5A) as well as incremented NSCs/Reactive-NSCs activation (entry into the cell cycle) (Figure 5B), and overall cell proliferation (Figures 5C,D), in the contralateral dentate gyrus (all measurements performed in the SGZ + GCL). Branching (Figures 5F,G), as well as thickening of the processes (Figures 5H,I), morphological hallmarks of React-NSCs, were significantly increased in aMTLE. Finally, we analyzed cell death in the SGZ + GCL, another hallmark of hippocampal sclerosis in MTLE, and found that it was significantly increased in the ipsilateral and the contralateral hippocampus of the aMTLE mice compared to the PBS-injected ones (Supplementary Figure 3). As in hcMTLE we found that in aMTLE reactive gliosis and induction of React-NSCs, as well as cell death, extended along the whole septo-temporal length of the dentate gyrus. Neuroblasts (DCX-positive cells) with abnormal morphology and location were found in both the ipsi and contralateral dentate gyrus (data not shown).

**FIGURE 6:**
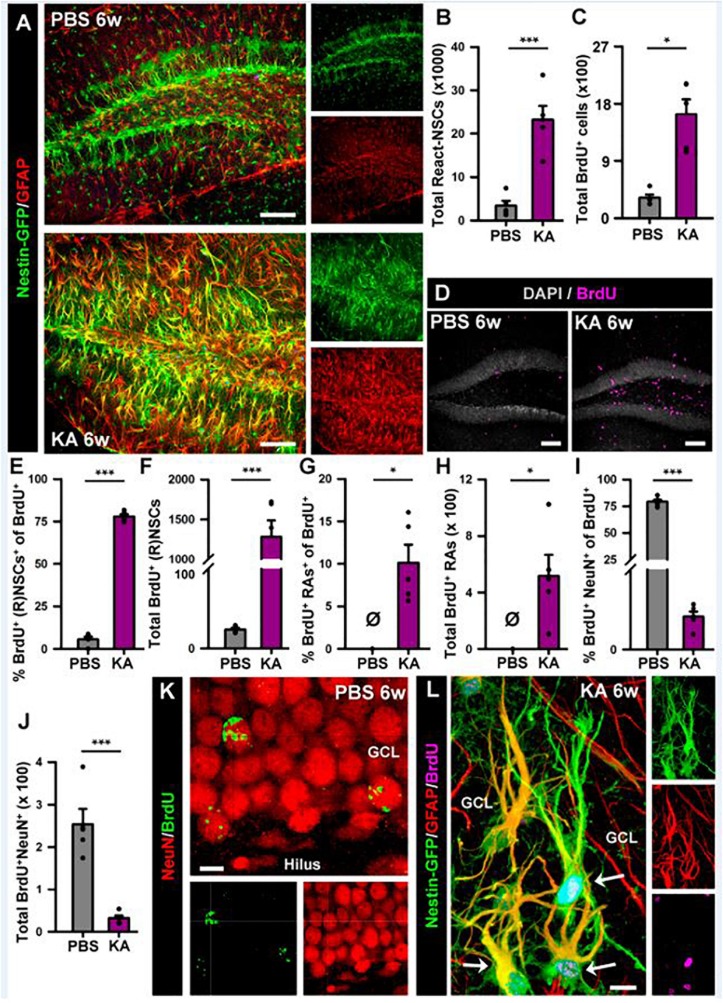
Intra-amygdalar injection of KA induces gliosis and React-NSCs in the dentate gyrus in the long term (6 weeks). BrdU (four injections, 2 h-apart) was injected the 2nd day after the KA injection. **(A)** Representative confocal microscopy images showing the reactive gliosis developed in the dentate gyrus. **(B)** Quantification of the number of React-NSCs (Nestin-GFP/GFAP-positive, S100ß-negative cells with reactive morphology). **(C)** Quantification of the number of BrdU-positive cells. **(D)** Representative confocal microscopy images for BrdU staining. **(E–I)** Quantifications of: **(E)** percentage of BrdU-labeled NSCs (in PBS) or React-NSCs (in KA) among total BrdU-positive cells; **(F)** Total number of BrdU-labeled NSCs/React-NSCs; **(G)** Percentage of BrdU-positive reactive astrocytes (BrdU/Nestin-GFP/S100ß-positive cells) among total BrdU-positive cells; **(H)** Total number of BrdU-labeled reactive astrocytes; **(I)** Percentage of BrdU-labeled NeuN-positive neurons among total BrdU-positive cells; **(J)** Total number of BrdU-labeled neurons. All Quantifications are referred to the SGZ + GCL. For quantifications for the hilus refer to Supplementary Figure 4. **(K)** Representative confocal microscopy image of BrdU-labeled neurons in the GCL of a control mouse. **(L)** Representative image of two BrdU-labeled React-NSCs (arrows) in the GCL of a KA mouse. Scale bar is 50 μm in **(A)** and **(D)**; and 10 μm in **(K)** and **(L)**. *n* = 5 in both groups. ^∗∗∗^*p* < 0.001, ^∗∗^*p* < 0.005, ^∗^*p* < 0.05 by Student’s *t*-test **(B,E)**; One-column sample *t*-test **(G,H)** and Mann–Whitney Rank Sum test **(C,I,J)**. Bars show mean ± SEM. Dots show individual data. The same settings for image acquisition were used for PBS and KA samples.

**FIGURE 7:**
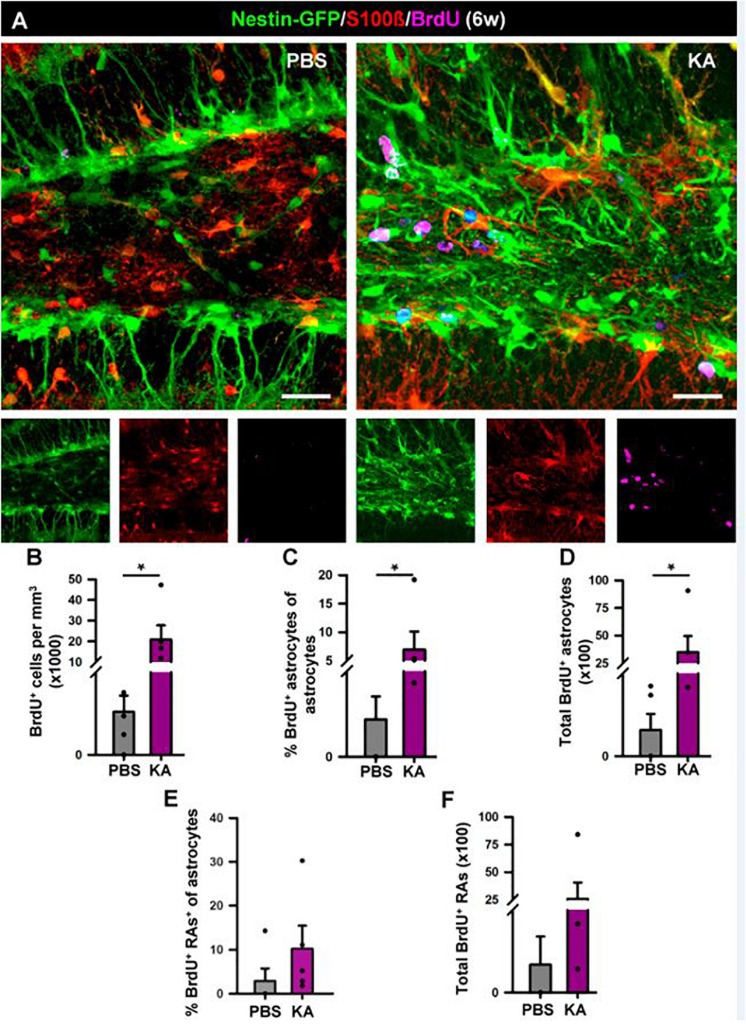
Development of seizure-reactive gliosis in the hilus in the longer term (6 weeks). **(A)** Representative confocal images of PBS (left panel) and KA (right) mice after staining for Nestin-GFP, BrdU, and S100ß to assess reactive gliosis. **(B–F)** Quantification of **(B)** density of BrdU^+^ cells; **(C)** Percentage of BrdU-labeled astrocytes among astrocytes; **(D)** Total number of BrdU-labeled astrocytes; **(E)** percentage of BrdU-labeled reactive astrocytes among astrocytes; **(F)** total BrdU-labeled reactive astrocytes. BrdU (four injections, 2 h-apart) was administered on the 2nd day after KA injection. Scale bar is 20 μm. *n* = 5 per group. ^∗^*p* < 0.05 by Mann–Whitney Rank Sum test. Bars show mean ± SEM. Dots show individual data.

In order to investigate the effects of seizures on the neurogenic niche in the longer term we studied animals that were sacrificed 6 weeks after the intra-amygdalar injection of KA (Figure 6). We first observed a marked gliosis in the dentate gyrus characterized by a massive generation of reactive astrocytes and/or React-NSCs. In sharp contrast with the PBS-injected mice, multibranched cells strongly expressing Nestin-GFP and GFAP were prominently distributed in the hilus, the SGZ, the GCL and to a lower extent into the molecular layer of the aMTLE mice (Figure 6A). The number of React-NSCs, described as Nestin-GFP/GFAP cells (with the morphological criteria explained before, Figure 2) was significantly increased in the KA mice (Figure 3B). We next quantified the number of BrdU cells (BrdU was administered in day 2 after the KA injection, the same as in the 1 w experiments) to assess differentiation. There were significantly more BrdU-positive cells in the SGZ + GCL of KA mice most likely reflecting the initial increase of cell division found at the 1-week time point (Figure 6C). BrdU-positive cells were also observed in the hilus (Figure 6D) where most of them were reactive astrocytes (Figure 7). We next assessed the BrdU-labeled population by cell types in the SGZ + GCL. The proportion of NSCs or React-NSCs in the KA mice, defined as Nestin-GFP (and GFAP)-positive cells lacking S100ß expression that were labeled with BrdU was significantly increased in the aMTLE animals (Figures 6E,L). As the total number of BrdU cells was increased, this translated in a significant increase in the total number of BrdU-labeled NSCs/React-NSCs (Figures 6F,L). BrdU-labeled reactive astrocytes (defined as S100ß and Nestin-GFP-positive cells) were absent in the control mice whereas they were abundant in the KA mice as observed by relative proportion over the total BrdU-positive population (Figure 6G) and in total number as quantified in the SGZ + GCL (Figure 6H). Reactive astrocytes were absent in the hilus of PBS-injected mice but were abundant in aMTLE mice (Figure 7). Due to the prominent reactive gliosis (Figure 7A) and the notorious increase in cell proliferation (Figure 7B) observed in the hilus the generation of astrocytes (Figures 7C,D) and of reactive astrocytes (Figures 7E,F) was assessed also in this region. A significant increase in astrogliogenesis and reactive astrogliogenesis was confirmed. Finally, we analyzed neurogenesis by colocalization of BrdU with the neuronal marker NeuN (Figure 6K). We found that neurogenesis was greatly impaired. The proportion of BrdU/NeuN-positive cells among the total BrdU population was significantly diminished (Figure 6I). In spite of the larger population of BrdU-positive cells, the total number of BrdU/NeuN-positive cells was also significantly decreased in the KA mice (Figure 3J). We checked also the expression of DCX, the specific marker of neuroblasts/immature neurons and confirmed the almost total absence of neurogenesis associated with reactive gliosis in the dentate gyrus (Supplementary Figures 4A,B). Noteworthy, the few DCX-positive cells that were found in the KA mice presented the morphological abnormalities that characterize aberrant neurogenesis (Supplementary Figures 4A,B). These results are very similar to those we reported for hcMTLE and show how the neuronal activity of the hippocampus and related structures is an essential regulator of NSC activity and how seizures cause a drastic disruption of the hippocampal neurogenic niche.

**FIGURE 8:**
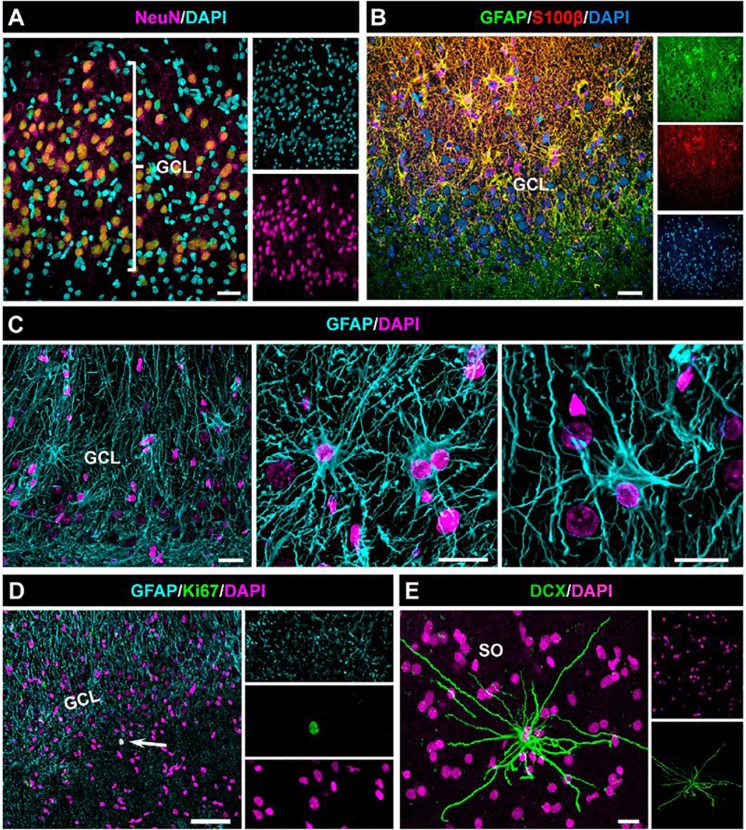
Absence of cell proliferation and neurogenesis in the hippocampus of drug-resistant MTLE patients. Unilateral resection of the hippocampus was performed for therapeutic purposes. Confocal microscopy images showing the characteristic dispersion of the GCL **(A)** and the reactive gliosis **(B)** which characterize MTLE. **(C)** Images of reactive astrocytes in the neurogenic niche. **(D)** Cell proliferation was extremely rare in the dentate gyrus. **(E)** A DCX-positive cell found in the *stratum oriens* (SO) of the hippocampus (CA3). No DCX-positive cell was found in the dentate of any of the samples. Scale bar is 15 μm in **(A)** and **(B)**; 20 μm in **(C)** and 10 μm in **(D)**.

We thus have observed in both aMTLE and the hcMTLE (Sierra et al., 2015) that reactive gliosis associates with abolished neurogenesis. We next investigated whether the same effect takes place in the human hippocampus. We analyzed hippocampi resected for therapeutic purposes from three patients (38, 46, and 56 years-old, all with hippocampal sclerosis ILAE type 1) of drug-resistant MTLE. We collected the samples as soon as they were removed from the patients to assure optimal conditions of tissue fixation. We studied the dentate gyrus and observed apparent granule cell dispersion (GCD) with the classical enlargement and loss of density of the GCL (Figure 8A). We also confirmed the presence of reactive gliosis using GFAP and S100β (Figure 8B) to identify astrocytes which presented the classical morphology of reactive astrocytes (Figure 8C). Importantly no cell resembling a putative NSC (radial morphology with soma located in the SGZ or lower part of the GCL) was found (Figure 8C). We cannot, however, make the claim that this result means that NSCs has transformed into React-NSC and ultimately into reactive astrocytes as reported to occur in the rodent models. We addressed the existence of cell division by using the mitosis marker Ki67. Ki67-positive cells were extremely rare in the dentate gyrus with none or just one cell found per human sample (Figure 8D). We also assessed the presence of neuroblasts (young migrating neurons) using immunostaining for DCX. No DCX-positive cell was found in the dentate gyrus of any of the human samples. In some samples a few scattered DCX-positive cells were found outside of the dentate gyrus (Figure 8E). The absence of cell proliferation and neurogenesis in the hippocampal neurogenic niche of patients of MTLE are agreement with what we have observed herein and in our previous study of hcMTLE (Sierra et al., 2015).

## Discussion

We had shown before that seizures induced in an experimental model of MTLE by intrahippocampal injection of MTLE have a profound effect on NSCs. Very soon after seizures NSCs become React-NSCs, i.e., they become multi branched with thicker processes overexpressing nestin and GFAP; lose their fine arborization in the molecular layer; migrate from the SGZ and closer to the molecular layer and get activated (enter mitosis) with much higher rate (Sierra et al., 2015). Finally, React-NSCs after several weeks become reactive astrocytes indistinguishable, at least by biomarker expression, morphology and location, to those derived from parenchymal astrocytes.

It could be argued that the injection of KA in hcMTLE could have a direct effect on NSCs. We now provide evidence that neuronal hyperexcitation of the hippocampal circuits triggered by the injection of KA in a separated, although connected, structure such as the amygdala provokes a very similar reactive reaction into the neurogenic niche of the dentate gyrus. 1 week after the intra-amygdalar injection of KA, the induction of React-NSCs with higher rate of activation and reactive gliosis were clear (Figure 2). In the longer term, 6 weeks after the injection of KA, the reactive gliosis was further developed and neurogenesis was almost abolished. The few neurons that were found presented the characteristic aberrant features (abnormal dendrites and location) described in other models of MTLE (Parent et al., 2006). Further, the aMTLE model allows further manipulations in the hippocampus. For instance we here present data from electrode arrays used to monitor neuronal activity over time to validate the model. In a similar fashion to hcMTLE (Bouilleret et al., 1999; Sierra et al., 2015), spontaneous seizures and epileptiform activity were recorded several weeks after the initial episodes of seizures triggered by the injection of KA suggesting that aMTLE mice become chronically epileptic.

The aMTLE model can be used in combination for instance with optogenetic manipulation and viral vector injections in the hippocampus without the surgery for the KA injection interfering with these manipulations. This work also provides confirmation for pro-gliogenic and anti-neurogenic effects of seizures in MTLE. Thus the cognitive functions associated with neurogenesis will be abolished or lost. Hippocampal neurogenesis is has been shown to participate in spatial and associative learning (Saxe et al., 2006; Dupret et al., 2008; Farioli-Vecchioli et al., 2008; Imayoshi et al., 2008; Clelland et al., 2009; Deng et al., 2009), as well as in the responses to stress and depression (Snyder et al., 2011). It could then be argued that part of the cognitive problems found in MTLE patients, such as memory impairment (Gargaro et al., 2013) and anxiety and depression (Heuser et al., 2009) as neuropsychiatric comorbidities could be attributed to impaired neurogenesis. We here show that the neurogenic niche of MTLE patients lacks cell proliferation and neurogenesis (Figure 4). A previous report, using similarly prepared samples, reported that cell proliferation (assessed by Ki67 staining) and neurogenesis (assessed by DCX immunostaining) are present in the adult human dentate gyrus and decline with age and could be similar or slightly lower in TLE (Fahrner et al., 2007). Although there has been intense debate regarding the presence of neurogenesis in the adult human hippocampus with reports in favor (Eriksson et al., 1998; Spalding et al., 2013; Boldrini et al., 2018) and against (Cipriani et al., 2018; Sorrells et al., 2018) a new report shows the existence of abundant neurogenesis in the adult, and even in the aged, human hippocampus by overcoming the technical obstacles associated with working with human tissue by tightly controlling for the *port-mortem* fixation conditions of the tissue (Moreno-Jiménez et al., 2019). An even newer work supports the persistence of cell proliferation and neurogenesis (again measured by DCX immunostaining) even in aged human brains (Tobin et al., 2019). One of the main articles arguing against the existence of adult human hippocampal neurogenesis uses epileptic tissue (Sorrells et al., 2018). Although in some experimental models of epilepsy boosted neurogenesis is found (Parent et al., 1997, 1998), longer-term studies reported depleted neurogenesis in rodents (Hattiangady et al., 2004) in accordance with data from humans (Mikkonen et al., 1998; Mathern et al., 2002; Pirttilä et al., 2005).

We conclude that NSCs respond swiftly to surrounding neuronal hyperexcitation and that when this activity is in the form of seizures, NSCs abandoned neurogenesis and switch to a reactive gliogenesis. Reactive gliosis, which have been proposed to be key to the development of secondary recurrent seizures (Devinsky et al., 2013) and neuroinflammation are being considered as targets for therapeutic efforts to fight epilepsy. Although the React-NSCs functional contribution to gliosis and neuroinflammation remains to be explored, because of the loss of neurogenesis, and arguably of its physiological functions and capacity to regenerate the dead neuronal population, we propose that React-NSCs should be considered targets as well.

## Data Availability

All datasets generated for this study are included in the manuscript and/or the Supplementary Files.

## Ethics Statement

Animals: All experiments for tissue analysis were done using the Nestin-GFP transgenic mouse line. All the animals were housed with *ad libitum* food and water access, in a 12:12 light:dark cycle. Nestin-GFP transgenic mouse line, kindly provided by Dr. Grigori Enikolopov at Cold Spring Harbor Laboratory (Cold Spring Harbor, NY, United States), were crossbred with C57BL/6 mice for at least 10 generations (Mignone et al., 2004). All procedures were approved by the University of the Basque Country (EHU/UPV) Ethics Committee (Leioa, Spain) and the Comunidad Foral de Bizkaia (CEEA: M20/2015/236). All procedures followed the European Directive 2010/63/EU and NIH guidelines. For electrophysiological recordings, all procedures involving animals were performed in accordance with the United States Public Health Service’s Policy on Humane Care and Use of Laboratory Animals, reviewed and approved by university (Comite Etico Cientifico para el Cuidado de Animales y Ambiente, CEC-CAA) and national (Comision Nacional de Investigacion Cientifica y Tecnologica, CONICYT) bioethics committees. Experiments were carried out with 2 months old mice (C57Bl/6 J) in accordance with the Ethics Committee (protocol CEBA 13-014). Human tissue: Human samples from individuals with MTLE. Freshly resected hippocampi from adult drug-resistant MTLE patients were obtained from the Basque Biobank at the Cruces University Hospital (Bilbao, Spain) with the patient’s written consent and with approval of the University of the Basque Country Ethics committee (CEISH/154/2012). The patient’s anonymity was preserved for this study. Sample MTLE030 corresponded to a 38-year-old male, MTLE049 to a 56-year-old male, and MTLE52 to a 46-year-old female. All of them with hippocampal sclerosis ILAE type 1.

## Author Contributions

TM-G participated in the experimental design, performed the experiments and data analysis, and prepared the figures regarding mouse tissue. SM-S participated in the experimental design, performed the experiments and data analysis, and prepared the figures regarding mouse and human tissue. NE participated in the experimental design, performed the experiments and data analysis, and prepared the figures regarding EEG recordings. RV-M participated in the experimental design, and performed the experiments and data analysis regarding mouse tissue. AM provided medical expertise on epilepsy and her participation was necessary for obtaining the human tissue. LZ, LG, and AS initiative and participation were necessary for obtaining the human tissue. PF participated in the overall project design and in experimental design, and performed the data analysis regarding the EEG recordings. JE designed the project, participated in the experimental design, performed the experiments and data analysis, prepared the figures, provided funding, and wrote the manuscript.

## Conflict of Interest Statement

The authors declare that the research was conducted in the absence of any commercial or financial relationships that could be construed as a potential conflict of interest.

## References

[B1] Altman J. Das G. D. (1965). Autoradiographic and histological evidence of postnatal hippocampal neurogenesis in rats. *J. Comp. Neurol.* 124 319–335. 10.1002/cne.901240303 5861717

[B2] Boldrini M. Fulmore C. A. Tartt A. N. Simeon L. R. Pavlova I. Poposka V. (2018). Human hippocampal neurogenesis persists throughout aging. *Cell Stem Cell* 22 589–599.e5. 10.1016/j.stem.2018.03.015 29625071 PMC5957089

[B3] Bouilleret V. Ridoux V. Depaulis A. Marescaux C. Nehlig A. Le Gal La Salle G. (1999). Recurrent seizures and hippocampal sclerosis following intrahippocampal kainate injection in adult mice: electroencephalography, histopathology and synaptic reorganization similar to mesial temporal lobe epilepsy. *Neuroscience* 89 717–729. 10.1016/s0306-4522(98)00401-1 10199607

[B4] Cipriani S. Ferrer I. Aronica E. Kovacs G. G. Verney C. Nardelli J. (2018). Hippocampal radial glial subtypes and their neurogenic potential in human fetuses and healthy and alzheimer’s disease adults. *Cereb. Cortex* 28 2458–2478. 10.1093/cercor/bhy096 29722804

[B5] Clelland C. D. Choi M. Romberg C. Clemenson G. D. Fragniere A. Tyers P. (2009). A functional role for adult hippocampal neurogenesis in spatial pattern separation. *Science* 325 210–213. 10.1126/science.1173215 19590004 PMC2997634

[B6] Deng W. Saxe M. D. Gallina I. S. Gage F. H. (2009). Adult born hippocampal dentate granule cells undergoing maturation modulate learning and memory in the brain. *J. Neurosci.* 29 13532–13542. 10.1523/JNEUROSCI.3362-09.2009 19864566 PMC2787190

[B7] Devinsky O. Vezzani A. Najjar S. De Lanerolle N. C. Rogawski M. A. (2013). Glia and epilepsy: excitability and inflammation. *Trends Neurosci.* 36 174–184. 10.1016/j.tins.2012.11.008 23298414

[B8] Dupret D. Revest J.-M. Koehl M. Ichas F. De Giorgi F. Costet P. (2008). Spatial relational memory requires hippocampal adult neurogenesis. *PLoS One* 3:e1959. 10.1371/journal.pone.0001959 18509506 PMC2396793

[B9] Encinas J. M. Enikolopov G. (2008). Identifying and quantitating neural stem and progenitor cells in the adult brain. *Methods Cell Biol.* 85 243–272. 10.1016/S0091-679X(08)85011-X 18155466

[B10] Encinas J. M. Michurina T. V. Peunova N. Park J.-H. Tordo J. Peterson D. A. (2011). Division-coupled astrocytic differentiation and age-related depletion of neural stem cells in the adult hippocampus. *Cell Stem Cell* 8 566–579. 10.1016/j.stem.2011.03.010 21549330 PMC3286186

[B11] Encinas J. M. Vaahtokari A. Enikolopov G. (2006). Fluoxetine targets early progenitor cells in the adult brain. *Proc. Natl. Acad. Sci. U.S.A.* 103 8233–8238. 10.1073/pnas.0601992103 16702546 PMC1461404

[B12] Eriksson P. S. Perfilieva E. Björk-Eriksson T. Alborn A.-M. Nordborg C. Peterson D. A. (1998). Neurogenesis in the adult human hippocampus. *Nat. Med.* 4 1313–1317. 10.1038/3305 9809557

[B13] Fahrner A. Kann G. Flubacher A. Heinrich C. Freiman T. M. Zentner J. (2007). Granule cell dispersion is not accompanied by enhanced neurogenesis in temporal lobe epilepsy patients. *Exp. Neurol.* 203 320–332. 10.1016/j.expneurol.2006.08.023 17049346

[B14] Farioli-Vecchioli S. Saraulli D. Costanzi M. Pacioni S. Cinà I. Aceti M. (2008). The timing of differentiation of adult hippocampal neurons is crucial for spatial memory. *PLoS Biol.* 6:e246. 10.1371/journal.pbio.0060246 18842068 PMC2561078

[B15] Gargaro A. C. Sakamoto A. C. Bianchin M. M. Geraldi Cde V. L. Scorsi-Rosset S. Coimbra ÉR. (2013). Atypical neuropsychological profiles and cognitive outcome in mesial temporal lobe epilepsy. *Epilepsy Behav.* 27 461–469. 10.1016/j.yebeh.2013.03.002 23611738

[B16] Hattiangady B. Rao M. Shetty A. (2004). Chronic temporal lobe epilepsy is associated with severely declined dentate neurogenesis in the adult hippocampus. *Neurobiol. Dis.* 17 473–490. 10.1016/j.nbd.2004.08.008 15571983

[B17] Heuser K. Taubøll E. Nagelhus E. A. Cvancarova M. Petter Ottersen O. Gjerstad L. (2009). Phenotypic characteristics of temporal lobe epilepsy: the impact of hippocampal sclerosis. *Acta Neurol. Scand.* 120 8–13. 10.1111/j.1600-0404.2009.01205.x 19566491

[B18] Huttmann K. Sadgrove M. Wallraff A. Hinterkeuser S. Kirchhoff F. Steinhauser C. (2003). Seizures preferentially stimulate proliferation of radial glia-like astrocytes in the adult dentate gyrus: functional and immunocytochemical analysis. *Eur. J. Neurosci.* 18 2769–2778. 10.1111/j.1460-9568.2003.03002.x 14656326

[B19] Imayoshi I. Sakamoto M. Ohtsuka T. Takao K. Miyakawa T. Yamaguchi M. (2008). Roles of continuous neurogenesis in the structural and functional integrity of the adult forebrain. *Nat. Neurosci.* 11 1153–1161. 10.1038/nn.2185 18758458

[B20] Indulekha C. L. Sanalkumar R. Thekkuveettil A. James J. (2010). Seizure induces activation of multiple subtypes of neural progenitors and growth factors in hippocampus with neuronal maturation confined to dentate gyrus. *Biochem. Biophys. Res. Commun.* 393 864–871. 10.1016/j.bbrc.2010.02.101 20171185

[B21] Jun H. Mohammed Qasim Hussaini S. Cho C. H. Welby J. Jang M.-H. (2015). Gadd45b mediates electroconvulsive shock induced proliferation of hippocampal neural stem cells. *Brain Stimul.* 8 1021–1024. 10.1016/j.brs.2015.07.036 26281755 PMC4656097

[B22] Mathern G. W. Leiphart J. L. De Vera A. Adelson P. D. Seki T. Neder L. (2002). Seizures decrease postnatal neurogenesis and granule cell development in the human fascia dentata. *Epilepsia* 43 68–73. 10.1046/j.1528-1157.43.s.5.28.x 12121298

[B23] Mikkonen M. Soininen H. Kälviäinen R. Tapiola T. Ylinen A. Vapalahti M. (1998). Remodeling of neuronal circuitries in human temporal lobe epilepsy: increased expression of highly polysialylated neural cell adhesion molecule in the hippocampus and the entorhinal cortex: remodeling of neuronal circuitries in TLE. *Ann. Neurol.* 44 923–934. 10.1002/ana.410440611 9851437

[B24] Moreno-Jiménez E. P. Flor-García M. Terreros-Roncal J. Rábano A. Cafini F. Pallas-Bazarra N. (2019). Adult hippocampal neurogenesis is abundant in neurologically healthy subjects and drops sharply in patients with Alzheimer’s disease. *Nat. Med.* 25 554–560. 10.1038/s41591-019-0375-9 30911133

[B25] Mouri G. Jimenez-Mateos E. Engel T. Dunleavy M. Hatazaki S. Paucard A. (2008). Unilateral hippocampal CA3-predominant damage and short latency epileptogenesis after intra-amygdala microinjection of kainic acid in mice. *Brain Res.* 1213 140–151. 10.1016/j.brainres.2008.03.061 18455706

[B26] Parent J. M. Elliott R. C. Pleasure S. J. Barbaro N. M. Lowenstein D. H. (2006). Aberrant seizure-induced neurogenesis in experimental temporal lobe epilepsy. *Ann. Neurol.* 59 81–91. 10.1002/ana.20699 16261566

[B27] Parent J. M. Janumpalli S. McNamara J. O. Lowenstein D. H. (1998). Increased dentate granule cell neurogenesis following amygdala kindling in the adult rat. *Neurosci. Lett.* 247 9–12. 10.1016/s0304-3940(98)00269-9 9637397

[B28] Parent J. M. Yu T. W. Leibowitz R. T. Geschwind D. H. Sloviter R. S. Lowenstein D. H. (1997). Dentate granule cell neurogenesis is increased by seizures and contributes to aberrant network reorganization in the adult rat hippocampus. *J. Neurosci.* 17 3727–3738. 10.1523/jneurosci.17-10-03727.1997 9133393 PMC6573703

[B29] Pirttilä T. J. Manninen A. Jutila L. Nissinen J. Kälviäinen R. Vapalahti M. (2005). Cystatin C expression is associated with granule cell dispersion in epilepsy. *Ann. Neurol.* 58 211–223. 10.1002/ana.20545 16049933

[B30] Saxe M. D. Battaglia F. Wang J.-W. Malleret G. David D. J. Monckton J. E. (2006). Ablation of hippocampal neurogenesis impairs contextual fear conditioning and synaptic plasticity in the dentate gyrus. *Proc. Natl. Acad. Sci. U.S.A.* 103 17501–17506. 10.1073/pnas.0607207103 17088541 PMC1859958

[B31] Segi-Nishida E. Warner-Schmidt J. L. Duman R. S. (2008). Electroconvulsive seizure and VEGF increase the proliferation of neural stem-like cells in rat hippocampus. *Proc. Natl. Acad. Sci. U.S.A.* 105 11352–11357. 10.1073/pnas.0710858105 18682560 PMC2516270

[B32] Seri B. García-Verdugo J. M. McEwen B. S. Alvarez-Buylla A. (2001). Astrocytes give rise to new neurons in the adult mammalian hippocampus. *J. Neurosci.* 21 7153–7160. 10.1523/jneurosci.21-18-07153.2001 11549726 PMC6762987

[B33] Sierra A. Martín-Suárez S. Valcárcel-Martín R. Pascual-Brazo J. Aelvoet S.-A. Abiega O. (2015). Neuronal hyperactivity accelerates depletion of neural stem cells and impairs hippocampal neurogenesis. *Cell Stem Cell* 16 488–503. 10.1016/j.stem.2015.04.003 25957904 PMC4443499

[B34] Snyder J. S. Soumier A. Brewer M. Pickel J. Cameron H. A. (2011). Adult hippocampal neurogenesis buffers stress responses and depressive behaviour. *Nature* 476 458–461. 10.1038/nature10287 21814201 PMC3162077

[B35] Song J. Zhong C. Bonaguidi M. A. Sun G. J. Hsu D. Gu Y. (2012). Neuronal circuitry mechanism regulating adult quiescent neural stem-cell fate decision. *Nature* 489 150–154. 10.1038/nature11306 22842902 PMC3438284

[B36] Sorrells S. F. Paredes M. F. Cebrian-Silla A. Sandoval K. Qi D. Kelley K. W. (2018). Human hippocampal neurogenesis drops sharply in children to undetectable levels in adults. *Nature* 555 377–381. 10.1038/nature25975 29513649 PMC6179355

[B37] Spalding K. L. Bergmann O. Alkass K. Bernard S. Salehpour M. Huttner H. B. (2013). Dynamics of hippocampal neurogenesis in adult humans. *Cell* 153 1219–1227. 10.1016/j.cell.2013.05.002 23746839 PMC4394608

[B38] Tobin M. K. Musaraca K. Disouky A. Shetti A. Bheri A. Honer W. G. (2019). Human hippocampal neurogenesis persists in aged adults and Alzheimer’s Disease patients. *Cell Stem Cell* 24 974–982. 10.1016/j.stem.2019.05.003 31130513 PMC6608595

