# Supplementary material for: Reactive Disruption of the Hippocampal Neurogenic Niche After Induction of Seizures by Injection of Kainic Acid in the Amygdala
Source: Front Cell Dev Biol. 2019 Aug 20;7:158. doi: 10.3389/fcell.2019.00158 (PMC6710991; doi:10.3389/fcell.2019.00158)
Supplement: Supplementary file 1 [file Data_Sheet_1.docx]

Reactive disruption of the hippocampal neurogenic niche after induction of seizures by injection of kainic acid in the amygdala.

**Supplementary Material and Methods**

**Animals:** All experiments for tissue analysis were done using the Nestin-GFP transgenic mouse line. All the animals were housed with *ad libitum* food and water access, in a 12:12 light:dark. Nestin-GFP transgenic mouse line, kindly provided by Dr. Grigori Enikolopov at Cold Spring Harbor Laboratory (Cold Spring Harbor, NY, USA), were crossbred with C57BL/6 mice for at least 10 generations (Mignone et al., 2004). All procedures were approved by the University of the Basque Country (EHU/UPV) Ethics Committee (Leioa, Spain) and the Comunidad Foral de Bizkaia (CEEA: M20/2015/236). All procedures followed the European directive 2010/63/UE and NIH guidelines. For electrophysiological recordings all procedures involving animals were performed in accordance to the U.S. Public Health Service’s Policy on Humane Care and Use of Laboratory Animals, reviewed and approved by university (Comite Ético Científico para el cuidado de animales y ambiente, CEC-CAA) and national (Comision Nacional de Investigacion Cientifica y Tecnologica, CONICYT) bioethics committees. Experiments were carried out with 2 months old mice (C57Bl/6 J) in accordance with the Ethics Committee (protocol CEBA 13-014).

**Intra-amygdalar injection**: Mice were anesthetized with intraperitoneal ketamine (Dechra Veterinary Products, Barcelona, Spain)/medetomidine (Braun VetCare, Tuttlingen, Germany) (75:1 mg/kg) and received a single dose of the analgesic buprenorphine (1mg/kg; Buprecare, Animalcare Lted, York, United Kingdom) subcutaneously. After positioning in the stereotaxic apparatus, a 0.8mm hole was drilled at coordinates taken from Bregma to target the right basolateral-amygdala: AP -1.4mm, LL -3.1mm and DV -4.7mm (Franklin and Paxinos 1997). A dose of 100 nL of sterile PBS or KA (Kainic acid monohydrate, Sigma, St Louis,MO, USA) at 12.96mM (1.3nmol) was injected to induce *status epilepticus* (Alves et al., 2017; Engel et al., 2017). After 2min, the microcapillar was retracted, and the mice sutured and maintained on a thermal blanket until recovered from anesthesia. This procedure is a modification of the one used in (Mouri et al., 2008). 5 mice were used in each group and time of analysis except for the 1week-PBS group in which 3 mice were analysed. Three mice were injected with fluorescein (CSFE) and sacrificed 24 h after to test the accuracy of the procedure (Fig.1).

**Electroencepaholgraphic recordings:** Neuronal activity was recorded by using a 32-channel silicon probe (Left hemisphere: A1×32-Poly2-5mm-50s-177; right hemisphere: 32 channel-4 shank silicon probe, Buzsáki 32 or A1x32-Edge-5mm-20-177 (Neuronexus, mean resistance 1MΩ.) stained with DiI (for a subsequent anatomical identification). The surgical procedure was performed following the methods described in (Bragin et al., 1997; Klee et al., 2017). Briefly, animals were induced with isoflurane (5%), and then anesthetized with urethane (initial dose: 0.8 g/kg dissolved in saline, i.p.), uniformly administered every 20–30 min by a programmable syringe pump (World Precision Instruments, model AL-1000) and placed in a stereotaxic device (Stoelting Co.). The level of anesthesia was assessed throughout the experiment by monitoring the reflex to tail pinch, respiratory rhythm, and the presence of low frequency LFP oscillations (~1 Hz). In case of increased excitability, an additional urethane dose (1/12 of the initial dose) was injected. Conversely, when signals of depression were detected, the pump was paused to allow the animal to recover from the deep sedation. During the entire experiment, body temperature was maintained at 36 ± 1°C using a homeothermic blanket (Harvard Apparatus, MA, USA) and monitored with a rectal probe connected to a temperature controller (Harvard Apparatus, MA, USA). Glucosaline solution was injected subcutaneously every 2 h. In the fully anesthetized mouse, the scalp was cut and retracted to expose the skull. Mouse was then implanted with a customized lightweight metal head holder and the head was held in a custom made metallic holder. Two small craniotomies (~1 mm each) were drilled on the skull over the recording sites. The stereotaxic coordinates, indicated by the stereotaxic atlas (Nita et al., 2007), were (relative to bregma): CA1 hippocampus, anteroposterior, −1.94 mm; mediolateral, +/−0.5 mm. Electrodes were positioned as close as possible to the dorsal CA1 stratum pyramidale, lowered via a motorized microdrive (~200 µm/min. Siskiyou, Grants Pass, OR, USA) to the recording positions dorsoventral 0.8–1 mm until ripple oscillations were visually detected online as hallmark for functional localization. An additional craniotomy (~500 µm) was made in the frontal bone of the left hemisphere to implant a metallic pin (500 µm diam. 3 mm length) as a recording reference. After lowering recording electrodes to recording positions, we waited for 5 minutes before starting recording sessions to allow brain tissue to settle in position. Electrical activity was recorded with an electrical amplifier (Intan RHD 2132 amplifier board connected to an RHD2000 evaluation system; Intan Technologies). Local field potential (LFP; sampling rate 20 kHz) were digitally filtered between 0.3 Hz–2 kHz. The same protocol was performed both in control (sham) and KA animals.

**BrdU administration**: BrdU (5-Bromo-1-(2-deoxy-β-D-ribofuranosyluracil, 5-Bromouracil deoxyriboside; Sigma, St Louis,MO, USA) was diluted in sterile saline and administered through intraperitoneal injections at 150 mg/kg concentration (15 mg/ml of BrdU was diluted in sterile phosphate-buffered saline (PBS) with 0.01N sodium hidroxyde(1% of the total solution). All the BrdU-injected mice received four injections separated by 2-h intervals on the second day after the injection of KA or PBS. Animals were sacrificed 1week or 6 weeks after the intraamygdalar injection of PBS or KA.

**Immunohistochemistry and cell quantification:** Experiments were performed essentially as described before following methods optimized for the use in transgenic mice (Encinas et al., 2006, 2011; Encinas and Enikolopov, 2008). Animals were deeply anesthetized and were subjected to transcardial perfusion with 25 ml of PBS followed by 30 ml of 4% (w/v) paraformaldehyde in PBS, pH 7.4. The brains were removed and post-fixed, with the same fixative solution, for 3 hr at room temperature, then transferred to PBS and kept at 4^o^C. Quantitative analysis of cell populations in transgenic mice was performed by means of design-based (assumption free, unbiased) stereology using a modified optical fractionator sampling scheme as previously described (Encinas and Enikolopov, 2008). Slices were collected using systematic-random sampling. The right hemisphere was selected per animal. The hemisphere was sliced sagittally in a lateral-to-medial direction, from the beginning of the lateral ventricle to the middle line, thus including the entire DG. The 50 μm slices (cut using a Leica VT 1200S vibrating blade microtome, Leica Microsystems GmbH, Wetzlar, Germany) were collected in 6 parallel sets, each set consisting of 12 slices, each slice 300 μm apart from the next. The sections were incubated with blocking and permeabilization solution (PBS containing 0.25% Triton-100X and 3% BSA) for 3hr at room temperature, and then incubated overnight with the primary antibodies (diluted in the same solution) at 4^o^C. After thorough washing with PBS, the sections were incubated with fluorochrome-conjugated secondary antibodies diluted in the blocking and permeabilization solution for 3 hr at room temperature. After washing with PBS, the sections were mounted on gelatin coated slides with DakoCytomation Fluorescent Mounting Medium (DakoCytomation, Carpinteria, CA). Those sections destined to the analysis of BrdU incorporation were treated, before the immunostaining procedure, with 2N HCl for 20 min at 37^o^C, rinsed with PBS, incubated with 0.1M sodium tetraborate for 10 min at room temperature, and then rinsed with PBS. The GFP signal from the transgenic mice was detected with an antibody against GFP for enhancement and better visualization. The following antibodies were used: chicken anti-GFP (Aves Laboratories, Tigard, OR) at 1:1000 dilution; rabbit anti-GFAP (Dako Cytomation) at 1:1000; rabbit anti-S100β (DakoCytomation) at 1:750; rat anti-BrdU (AbD Serotech, Kidlington, UK) at 1:1000, rabbit anti Ki67 (DBS Pleasanton, CA USA); rabbit anti-NeuN (Abcam) at 1:500; rabbit anti-Tbr1 (Cambridge, England) at 1:1000; goat anti-DCX (St Cruz) at 1:500; AlexaFluor 488 goat anti-chicken (Molecular Probes, Willow Creek Road, Eugene, OR) at 1:500; AlexaFluor 647goat anti-rabbit (Molecular Probes) at 1:500; AlexaFluor 568 goat anti-rat (Molecular Probes) at 1:500; AlexaFluor 568 Donkey anti-goat (Molecular Probes) at 1:500; DAPI, at 1:1000 (Sigma) was used at counterstaining when required. All BrdU cells per slice were counted at 40X magnification in a set of slices. Then in different sets of slices BrdU-positive cells were categorized with different combination of antibodies (Nestin-GFP/GFAP; Nestin-GFP/S100β; Nestin-GFP/NeuN) at 63x. In randomly selected fields (by starting at the lateral tip of the upper blade of the GCL and then skipping 2 field and imaging the 3rd, the 6^th^ etc., following the GCL (upper and lower blade) along the dentate gyrus, 50 to 100 BrdU cells per animal were categorized as NSCs, React-NSCs, neurons, astrocytes, or reactive astrocytes following the criteria described previously (Encinas et al., 2006, 2011; Encinas and Enikolopov, 2008; Sierra et al., 2015). NSCs were defined as radial glia-like cells positive for Nestin-GFP and GFAP with the soma located in the SGZ or the lower third of the GCL and with a process extending from the SGZ towards the molecular layer through the GCL. Cells devoid of GFAP immunostaining and with none or short horizontal processes. Neurons were identified by NeuN immunostaining. Astrocytes were identified as GFAP- or S100β-positive cells, negative for Nestin-GFP staining, with stellar morphology. Reactive astrocytes were defined as Nestin- GFP- positive cells immunopositive also S100β and with hypertrophic soma, thickened processes and star-like morphology. The relative proportions of BrdU-positive cell types were referred to the total number of BrdU to estimate the absolute number of each BrdU cell type population. Those cells in the uppermost focal plane were not counted to avoid overestimation. To measure the morphological changes in React-NSCs, at least 50 cells were randomly selected from 20 μm-thick z-stacks taken from PBS and aMTLE mice brain sections immunostained for GFP and GFAP. The quantification was performed in flat projections generated from the z-stacks. Primary processes were considered to be those emerging directly from the soma, and the secondary processes those emerging from the primary processes in the 45 μm closest to the soma. The thickness of the primary process was measured at two points: at 5 μm from the soma and at 15 μm from the soma. Quantifications were performed separately for the SGZ+GCL and for the hilus. The immunostaining protocol was the same for mouse and human samples. For quantification of cell death we followed the criteria described in (Abiega et al., 2016; Sierra et al., 2010). Apoptosis is characterized by specific nuclear morphology such as pyknosis (DNA condensation) and karyorrhexis (nuclear fragmentation), both detected by condensed staining with DAPI in 63x randomly selected fields as described above.

**Image capture:** All fluorescence immunostaining images were collected employing a Leica SP8 (Leica, Wetzlar, Germany) laser scanning microscope and their corresponding manufacturer’s software following protocols optimized for stereotaxic quantification and quantitative image analysis (Encinas et al., 2011; Sierra et al., 2015).

The signal from each fluorochrome was collected sequentially, and controls with sections stained with single fluorochromes were performed to confirm the absence of signal leaking into different channels and antibody penetration. Settings were the same for PBS and KA mice in each time point of analysis and for each magnification used. All images were imported into Adobe Photoshop 7.0 (Adobe Systems Incorporated, San Jose, CA) in tiff format. Brightness, contrast, and background were adjusted equally for the entire image using the “levels” controls from the “image/adjustment” set of options without any further modification. Al images shown are projections from z-stacks ranging from 10 (typically for individual cell images) to 20 μm of thickness.

**Human tissue:** Human samples from individuals with MTLE. Freshly resected hippocampi from adult drug-resistant MTLE patients were obtained from the Basque Biobank at the Cruces University Hospital (Bilbao, Spain) with the patient’s written consent and with approval of the University of the Basque Country Ethics committee (CEISH/154/2012). The patient´s anonymity was preserved for this study. Sample MTLE030 corresponded to a 38 year-old male; MTLE049 to a 56 year-old male and MTLE52 to a 46 year-old female. All of them with hippocampal sclerosis ILAE type 1. Immediately after surgery, the tissue was immersed in saline and maintained refrigerated until transported to the Pathology Unit of the Hospital (under 40min), where it was manually sectioned in 1- 2mm thick coronal sections and transferred to 4% paraformaldehyde (PFA) in PBS, pH 7.4 for 30min, then washed in PBS. Samples were sliced into 50-μm slices using a Leica VT 1200S vibrating blade microtome and immunostained for Ki67; NeuN, GFAP, DCX and S100β and co-stained with DAPI as described before. The same antibodies and concentrations were used for mouse and human tissue.

**Statistical analysis:** SigmaPlot (San Jose, CA, USA) was used for statistical analysis.

For the analysis of pairs of groups when the data passed the normality test a Student ́s t test was performed. When the data did not pass the normality test a non-parametric Mann-Whitney Rank Sum Test was performed. For the analysis of pairs of groups when one of the conditions presented 0 as values, a one-sample t test against a hypothetical value of 0 was performed. Only p<0.05 is reported to be significant. Data are shown as mean ± standard error of the mean (SEM).

**Supplementary Figures**

**
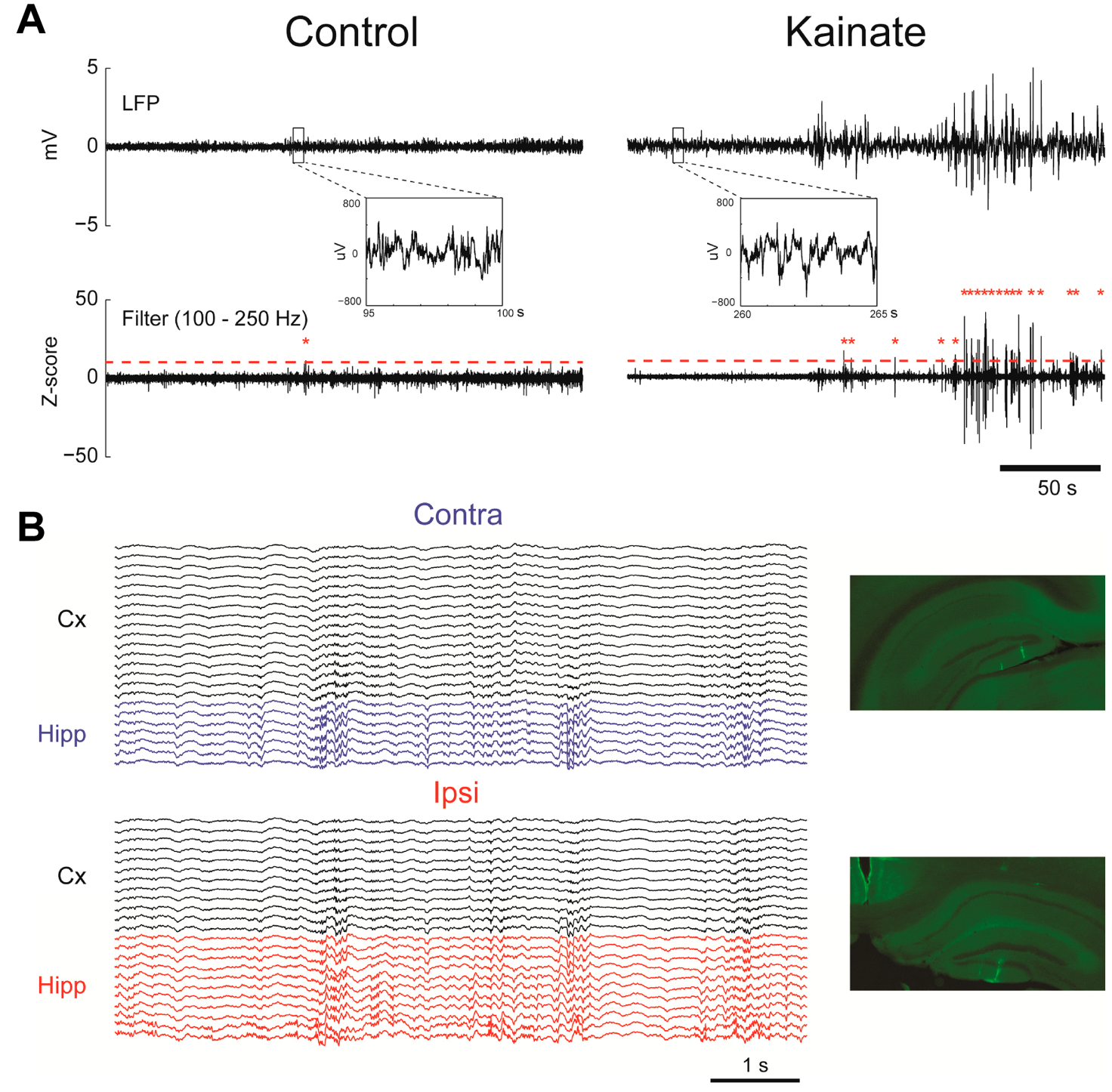
**

**Supplementary Figure 1. Electrophysiological recordings. (A)** Automatic detection for epileptiform events. Example recordings from control (Left) and KA (Right) animals. Raw LFP (Top) were band-pass filtered between 100–250 Hz and Z-scored (Bottom) to detect events exceeding a threshold of 10 SD (dashed red line). Red asterisks indicate potential epileptiform events surpassing the threshold. To evaluate the robustness of the automatic detection, the threshold was set to more restrictive (higher) values and the statistical result was maintained for the overall epileptiform event frequency. Insets depict the typical electric activity under anesthesia during baseline conditions. **(B)** Multichannel activity during baseline conditions for recording shown in Fig. 1. For the hemisphere contralateral to the kainate injection (Top panel) electrodes were spaced at 20 microns intervals in the mouse cortex (Cx, black) and hippocampus (Hipp, blue). The fluorescent microscopy photomicrograph depicts the final location of the probe in this experiment (Right) at the level of the dentate gyrus. Ipsilateral hemisphere (Bottom panel) display a similar activity, with electrodes spaced at 25 microns.


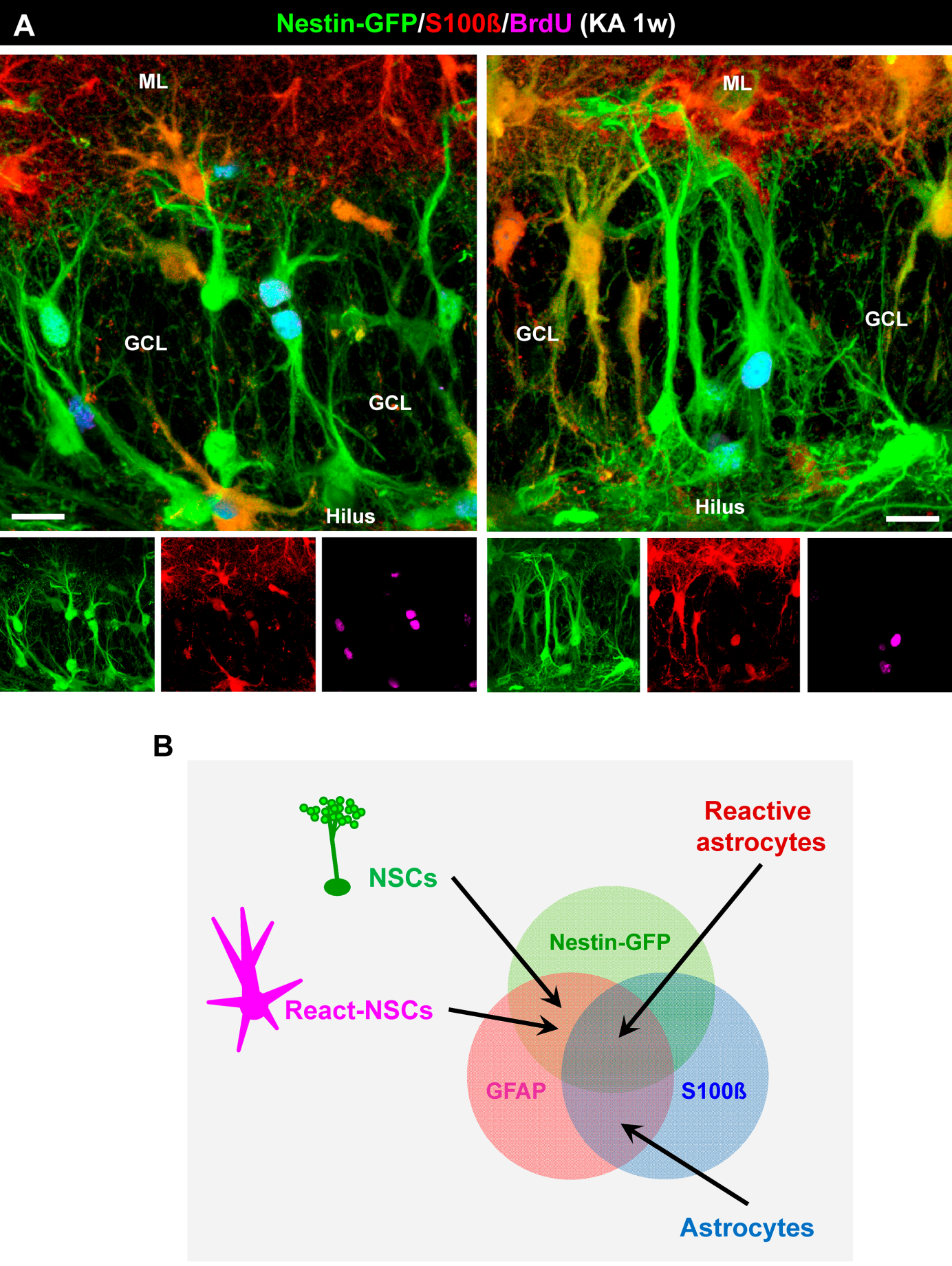


**Supplementary Figure 2. Seizure-induced generation of reactive astrocytes in the neurogenic niche. (A)** Representative confocal microscopy images of the hippocampus after costaining with Nestin-GFP; BrdU and S100β, 1 week after the intramygdalar injection of KA (5 days after administration of BrdU, 4 injections, 2h-apart) BrdU-labeled Nestin-GFP cells with expression of S100β, a biomarker of mature astrocytes were found in the GCL. **(B)** NSCs, React-NSCs and reactive astrocytes express Nestin-GFP and GFAP. Reactive astrocytes but not NSCs or React-NSCs express S100β. Astrocytes express GFAP and S100β but not Nestin-GFP. React-NSCs are morphologically different from NSCs.

**
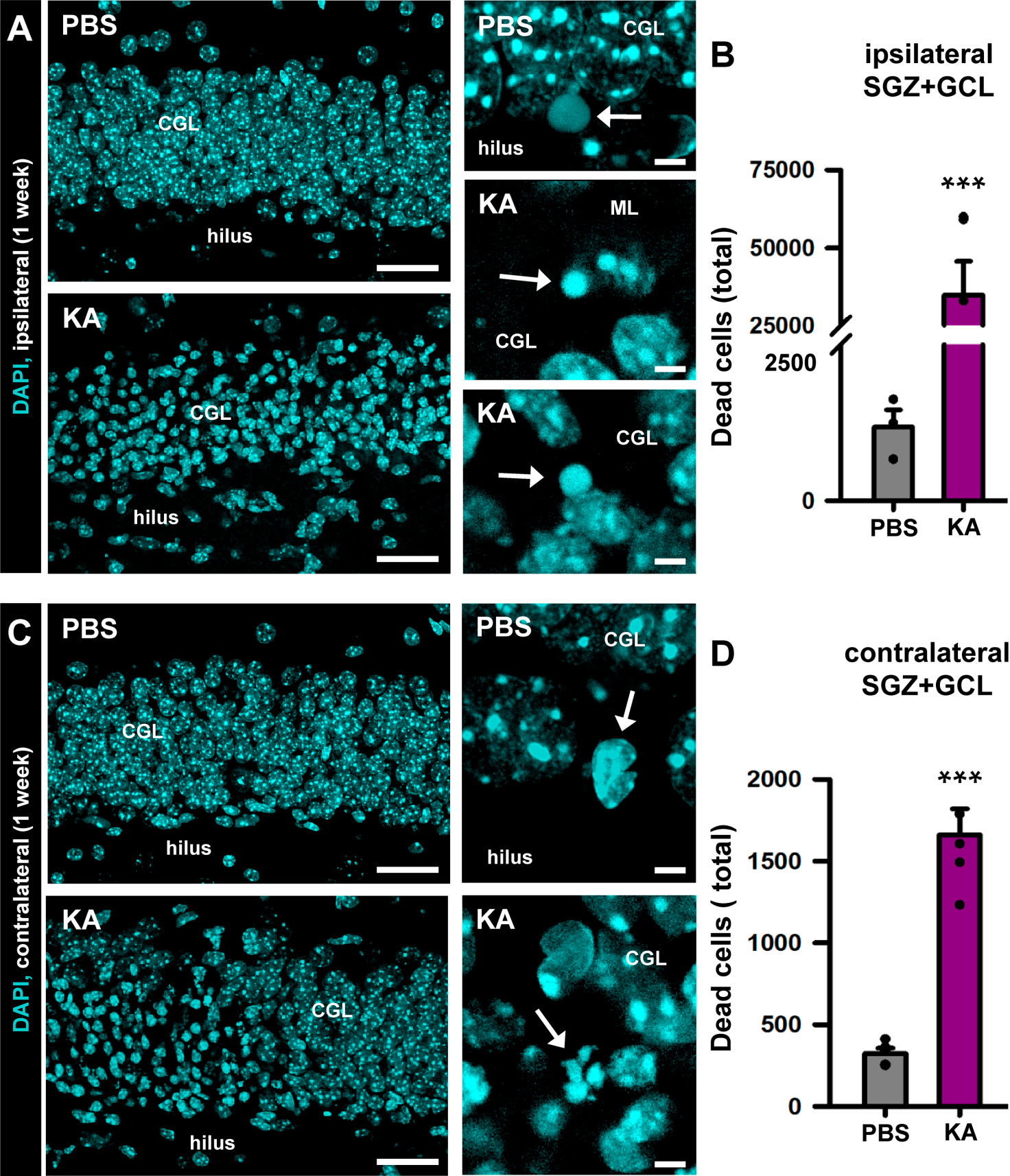
**

**Supplementary Figure 3. Cell death in the ipsi and contrlateral dentate gyrus of aMTLE mice.** Cell death was quantified by identifying nuclei presenting pyknosis (DNA condensation) and/or karyorrhexis (nuclear fragmentation), both detected by condensed staining with DAPI. **(A)** Representative confocal microscopy images, after staining for DAPI, showing the SGZ+GCL in the hippocampus ipsilateral to the PBS or KA injection in the amygdala. Examples of pyknotic/karyorrhexic nuclei are also shown at higher magnification. **(B)** Quantifcation of the total number of death cells in the ipsilateral hippocampus (SGZ+GCL). **(C)** Representative confocal microscopy images, after DAPI staining, showing the SGZ+GCL in hippocampus contralateral to the PBS or KA injection in the amygdala. Examples of pyknotic/karyorrhexic nuclei are also shown at higher magnification. **(D)** Quantifcation of the total number of death cells in the contralateral hippocampus (SGZ+GCL). n = 5 per group. Scale bar is 10 μm in the lower magnification photographs and 5 μm in the higher magnification ones. ***p < 0.001.

**
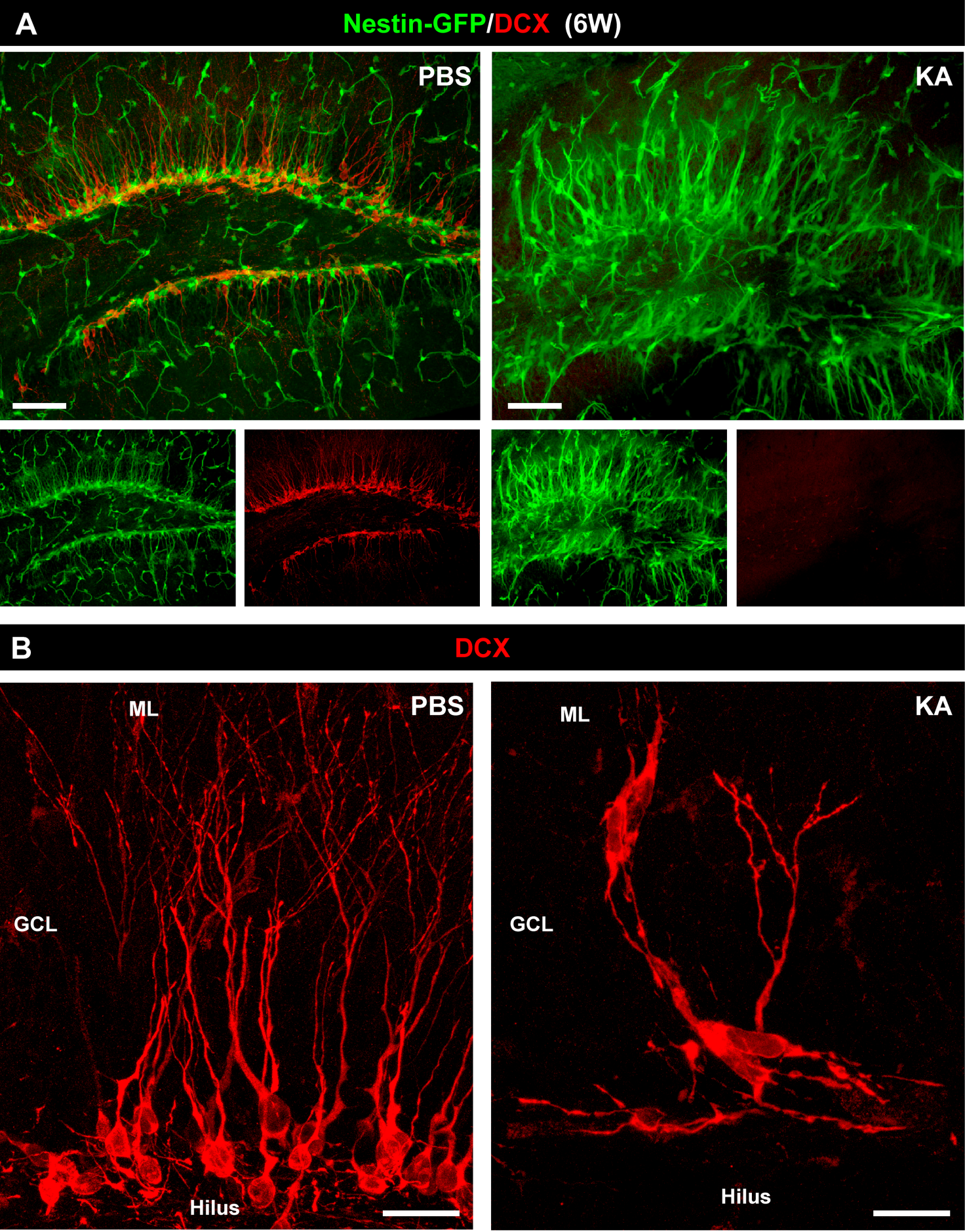
**

**Supplementary Figure 4. Seizures induce the loss of neurogenesis in the longer term.** **(A)** Staining for DCX, an specific biomarker for neuroblasts and young neurons showed that neurogenesis was abundant in the PBS-injected mice (left panel) but was mostly absent in the dentate gyrus of the KA-injected mice (right panel). **(B)** Those few neuroblasts that could be found presented aberrant morphology and location.

**Supplementary References**

Abiega, O., Beccari, S., Diaz-Aparicio, I., Nadjar, A., Layé, S., Leyrolle, Q., et al. (2016). Neuronal Hyperactivity Disturbs ATP Microgradients, Impairs Microglial Motility, and Reduces Phagocytic Receptor Expression Triggering Apoptosis/Microglial Phagocytosis Uncoupling. *PLoS Biol.* 14, e1002466. doi:10.1371/journal.pbio.1002466.

Alves, M., Gomez-Villafuertes, R., Delanty, N., Farrell, M. A., O’Brien, D. F., Miras-Portugal, M. T., et al. (2017). Expression and function of the metabotropic purinergic P2Y receptor family in experimental seizure models and patients with drug-refractory epilepsy. *Epilepsia* 58, 1603–1614. doi:10.1111/epi.13850.

Bragin, A., Penttonen, M., and Buzsáki, G. (1997). Termination of Epileptic Afterdischarge in the Hippocampus. *J. Neurosci.* 17, 2567–2579. doi:10.1523/JNEUROSCI.17-07-02567.1997.

Encinas, J. M., and Enikolopov, G. (2008). “Identifying and Quantitating Neural Stem and Progenitor Cells in the Adult Brain,” in *Methods in Cell Biology* (Elsevier), 243–272. doi:10.1016/S0091-679X(08)85011-X.

Encinas, J. M., Michurina, T. V., Peunova, N., Park, J.-H., Tordo, J., Peterson, D. A., et al. (2011). Division-coupled astrocytic differentiation and age-related depletion of neural stem cells in the adult hippocampus. *Cell Stem Cell* 8, 566–579. doi:10.1016/j.stem.2011.03.010.

Encinas, J. M., Vaahtokari, A., and Enikolopov, G. (2006). Fluoxetine targets early progenitor cells in the adult brain. *2006*, 6.

Engel, T., Martinez-Villarreal, J., Henke, C., Jimenez-Mateos, E. M., Sanz-Rodriguez, A., Alves, M., et al. (2017). Spatiotemporal progression of ubiquitin-proteasome system inhibition after status epilepticus suggests protective adaptation against hippocampal injury. *Mol Neurodegener* 12, 21. doi:10.1186/s13024-017-0163-2.

Franklin KBJ, Paxinos G. (1997). *The Mouse Brain in Stereotaxic 10.1111/j.1469-7580.2004.00264.x.* San Diego: Academic Press.

Klee, R., Brandt, C., Töllner, K., and Löscher, W. (2017). Various modifications of the intrahippocampal kainate model of mesial temporal lobe epilepsy in rats fail to resolve the marked rat-to-mouse differences in type and frequency of spontaneous seizures in this model. *Epilepsy Behav* 68, 129–140. doi:10.1016/j.yebeh.2016.11.035.

Mignone, J. L., Kukekov, V., Chiang, A.-S., Steindler, D., and Enikolopov, G. (2004). Neural stem and progenitor cells in nestin-GFP transgenic mice. *The Journal of Comparative Neurology* 469, 311–324. doi:10.1002/cne.10964.

Mouri, G., Jimenez-Mateos, E., Engel, T., Dunleavy, M., Hatazaki, S., Paucard, A., et al. (2008). Unilateral hippocampal CA3-predominant damage and short latency epileptogenesis after intra-amygdala microinjection of kainic acid in mice. *Brain Research* 1213, 140–151. doi:10.1016/j.brainres.2008.03.061.

Nita, D. A., Cissé, Y., Timofeev, I., and Steriade, M. (2007). Waking-sleep modulation of paroxysmal activities induced by partial cortical deafferentation. *Cereb. Cortex* 17, 272–283. doi:10.1093/cercor/bhj145.

Sierra, A., Encinas, J. M., Deudero, J. J. P., Chancey, J. H., Enikolopov, G., Overstreet-Wadiche, L. S., et al. (2010). Microglia shape adult hippocampal neurogenesis through apoptosis-coupled phagocytosis. *Cell Stem Cell* 7, 483–495. doi:10.1016/j.stem.2010.08.014.

Sierra, A., Martín-Suárez, S., Valcárcel-Martín, R., Pascual-Brazo, J., Aelvoet, S.-A., Abiega, O., et al. (2015). Neuronal Hyperactivity Accelerates Depletion of Neural Stem Cells and Impairs Hippocampal Neurogenesis. *Cell Stem Cell* 16, 488–503. doi:10.1016/j.stem.2015.04.003.
